# *Bartonella vinsonii* subsp. *arupensis* in Humans, Thailand

**DOI:** 10.3201/eid1806.111750

**Published:** 2012-06

**Authors:** Ying Bai, Michael Y. Kosoy, Maureen H. Diaz, Jonas Winchell, Henry Baggett, Susan A. Maloney, Sumalee Boonmar, Saithip Bhengsri, Pongpun Sawatwong, Leonard F. Peruski

**Affiliations:** Centers for Disease Control and Prevention, Fort Collins, Colorado, USA (Y. Bai, M.Y. Kosoy);; Centers for Disease Control and Prevention, Atlanta, Georgia, USA (M.H. Diaz, J. Winchell);; Centers for Disease Control and Prevention, Nonthaburi, Thailand (H. Baggett, S.A. Maloney, S. Boonmar, S. Bhengsri, P. Sawatwong, L.F. Peruski)

**Keywords:** Bartonella vinsonii subsp. arupensis, animal hosts, Thailand, undiagnosed diseases, bacteria, zoonoses

## Abstract

We identified *Bartonella vinsonii* subsp. *arupensis* in pre-enriched blood of 4 patients from Thailand. Nucleotide sequences for transfer-messenger RNA gene, citrate synthase gene, and the 16S–23S rRNA internal transcribed spacer were identical or closely related to those for the strain that has been considered pathogenic since initially isolated from a human in Wyoming, USA.

More than 30 species of bartonellae that are highly prevalent in a wide variety of vertebrates have been described. *Bartonella bacilliformis*, *B. henselae*, and *B. quintana* are well-known human pathogens, and several other *Bartonella* species, including *B. elizabethae*, *B. tamiae*, *B. vinsonii* subsp. *arupensis*, have been associated with various clinical manifestations in humans (*1**–**3*). A link between some of these pathogenic strains and their animal hosts has been documented; for example, *B. elizabethae* is linked with *Rattus* rats. However, the reservoir host is unknown for other species, such as *B. tamiae*, which was isolated in patients from Thailand (*2*).

*B. vinsonii* subsp. *arupensis* was first isolated from a bacteremic cattle rancher in Wyoming, USA, in 1999 (*3*). Later studies showed that strains identical to *B. vinsonii* subsp. *arupensis* were highly prevalent among deer mice (*Peromyscus maniculatus*), a strictly North American rodent species frequently found across a wide geographic area, including Wyoming. Similar strains of *B. vinsonii* subsp. *arupensis* have not been found in other animals in North America, suggesting that deer mice are natural hosts of this bacterium (*4*).

However, the proposed link between infected mice and *B. vinsonii* subsp. *arupensis* infection in humans was challenged when this bacterium was reported in an endocarditis patient in France (*5*) and 2 febrile patients in Russia (*6*). The link was further disputed after identification of *B. vinsonii* subsp. *arupensis* infection in 2 humans in Thailand (*7*) and the subsequent inability to identify this strain or related species among the local rodent population, despite intensive investigation in different parts of Thailand (*8*). *B. vinsonii* subsp. *arupensis* was also identified in stray dogs in Thailand (*9*). In addition, *B. vinsonii* subsp. *arupensis*–specific antibodies were reported in febrile patients from Nepal (*10*). Together, these reports suggest that the spectrum of animal hosts carrying *B. vinsonii* subsp. *arupensis* may be underestimated. We report the identification of *B. vinsonii* subsp. *arupensis* in 4 more patients in Thailand.

## The Study

The patients were enrolled in a febrile illness study in 4 rural hospitals in Chiang Rai and Khon Kaen Provinces, in northern and northeastern Thailand, respectively, during February 2002–March 2003. One of the patients (no. 45-00250) was enrolled as an afebrile patient. The 4 patients had some common clinical symptoms, such as headache, myalgias, dizziness, and fatigue. In addition, 3 of the patients had elevated levels of liver enzymes. All patients reported trapping or killing rats or seeing rats inside or around their houses during the 2 weeks before onset of symptoms, and all patients owned a dog and/or cat (Table).

**Table:** Clinical characteristics and animal exposures of 4 patients with *Bartonella vinsonii* subsp. *arupensis* detected in blood, Thailand, February 2002–March 2003*

Patient ID no.	Age, y/sex	Occupation	Province	Clinical symptoms	Abnormal laboratory test results	Pet(s)
45–00025	56/M	Farmer	Chiang Rai	Fever, chill, fatigue, myalgia	AST 70 IU/dL, ALP 210 IU/dL	Cat
45–01217	11/F	Student	Khon Kaen	Fever, chill, fatigue, myalgia, headache, icterus	Hb 11.3 g/dL, ALP 663 IU/dL	Cat, dog
45–01239	39/F	Business	Khon Kaen	Fever, chill, fatigue, myalgia, headache, eye pain	Hb 12.1 g/dL, AST 73 IU/dL, ALT 141 IU/dL, ALP 388 IU/dL	Dog
45–00250	35/M	Day laborer	Khon Kaen	Fatigue, myalgia, joint pain, headache, cough, abdominal pain	Normal values	Dog

*All patients reported exposure to rats during the 2 weeks before onset of symptoms. ID, identification; AST, aspartate aminotransferase (reference value 5–35 IU/dL); ALP, alkaline phosphatase (reference value 30–115 IU/dL); Hb, hemoglobin (reference value <13.8 g/dL); ALT, alanine aminotransferase (reference value 5–35 IU/dL).

To determine if the patients were infected with *Bartonella* species, we tested blood clots from each patient for the presence of the bacterium. For testing, we used a liquid growth medium (*Bartonella* α-*Proteobacteria* growth medium [BAPGM]) as a pre-enrichment step (*11*), improved molecular assays for *Bartonella* detection (*12*), and established molecular methods. The blood clots were inoculated into freshly prepared BAPGM. To avoid potential contamination, we did not supplement the medium with animal blood. Blank BAPGM controls were included for each inoculation. The inoculants and the blank controls were incubated aerobically at 35°C in 5% CO_2_ for 7 days.

DNA was extracted from this pre-enriched medium by using the QIAamp DNA Mini Kit (QIAGEN, Valencia, CA, USA). DNA was analyzed following published protocols (*12**–**14*). In brief, real-time PCR, nested PCR, and conventional PCR were used to target *Bartonella*-specific regions in the transfer-messenger RNA (*ssrA*) gene, the citrate synthase (*gltA*) gene, and the 16S–23S rRNA internal transcribed spacer, respectively. Positive controls (*B. doshiae*) and negative controls were included within each PCR run to evaluate the presence of appropriately sized amplicons and contamination, respectively. Amplicons were recovered from PCRs by gel purification (QIAGEN) and sequenced in both directions.

Sequences for the 3 targets were similar to those for the type strain of *B. vinsonii* subsp. *arupensis*. The *gltA* sequences (338 bp) from all 4 patient samples were identical to those for previously reported variants from a febrile patient in Thailand (GenBank accession no. GQ200857) and from stray dogs in Thailand (GenBank accession no. FJ946836). These *gltA* sequences were 0.8% divergent from the type strain of *B. vinsonii* subsp. *arupensis* (GenBank accession no. AF214557).

The *ssrA* sequences (251 bp) revealed 2 similar variants. One variant was identical to the type strain of *B. vinsonii* subsp. *arupensis* (GenBank accession no. JN029783) and was identified in samples from 3 of the patients (45-00250, 45-01217, and 45-01239). The other variant (GenBank accession no. JN394654), from patient 45-00025, was 2.8% divergent from the type strain of *B. vinsonii* subsp. *arupensis.*

In addition, two 16S–23S rRNA internal transcribed spacer variants were identified. One variant (GenBank accession no. JN402327), identified in samples from 3 patients (45-00025, 45-01217, and 45-01239), was 0.4% divergent from the type strain of *B. vinsonii* subsp. *arupensis* (GenBank accession no. AF312504). The other variant (GenBank accession no. JN402328), from patient 45-00250, was 0.9% divergent from the type strain of *B. vinsonii* subsp. *arupensis* (GenBank accession no. AF312504).

## Conclusions

In Thailand, at least 2 *Bartonella* species, *B. henselae* and *B. tamiae,* have been reported in association with human diseases (*2**,**15*). In addition, *Bartonella* DNA was detected recently in acutely ill patients from Thailand (*7*). *gltA* sequences revealed a broad range of *Bartonella* species in these patients, including *B. elizabethae*, *B. tribocorum*, *B. rattimassiliensis*, and *B. vinsonii* subsp. a*rupensis*. This finding suggests that numerous species of *Bartonella* may be associated with acute illness in Thailand.

In the current study, sequence data from 3 genetic targets provide additional evidence to confirm infection with *B. vinsonii* subsp. *arupensis* in patients in Thailand. Although the potential source(s) and mechanism(s) of transmission of this bacterium to humans remain unclear, the previous finding of *B. vinsonii* subsp. *arupensis* among stray dogs in Thailand (*9*) and the fact that 3 of the 4 patients in this report owned dogs could suggest dogs might be a source of the bacterium. Commonality of rat exposure may suggest that rats can also be potential reservoir hosts of *B. vinsonii* subsp. *arupensis*. Further investigation is needed in this regard.

It is known that diseases of humans and animals can translocate across the globe. For example, bubonic plague, caused by *Yersinia pestis*, was spread by infected rats on ships traveling from Asia to other continents. *Bartonella elizabethae* and other rat-associated *Bartonella species* that originated in the Old World have been similarly translocated to the New World and other regions (*1**,**8*). Reports of infections caused by *B. vinsonii* subsp. *arupensis* in humans in France, Russia, and Nepal, suggest that the role of this bacterium as a human pathogen may be more geographically widespread than previously believed.

Given the postulated New World origin of this pathogen, its ecology might serve as a model system to examine a possible translocation of *Bartonella* species across broad geographic regions and even between hosts. In addition, our finding of *B. vinsonii* subsp. *arupensis* in patients in Thailand suggests that this pathogen may be responsible for some undiagnosed cases of febrile illness and other types of illnesses in Thailand and possibly other countries in Asia.
